# High-dose intravenous hydromorphone for patients who use opioids in the hospital setting: time to reduce the barriers

**DOI:** 10.1186/s12954-021-00533-0

**Published:** 2021-08-13

**Authors:** Laura E. Labonté, Samantha Young

**Affiliations:** 1 Department of Psychiatry, University of British Columbia, Vancouver, BC Canada; 2 Interdepartmental Division of Addiction Medicine, St. Paul’s Hospital, Vancouver, BC Canada; 3 Institute of Health Policy, Management and Evaluation, Dalla Lana School of Public Health, University of Toronto, Toronto, ON Canada; 4 Division of General Internal Medicine, Department of Medicine, St. Michael’s Hospital, Unity Health, 30 Bond Street, Toronto, ON Canada

**Keywords:** Intravenous administration, Opiate substitution treatment, Hydromorphone, Hospitalization

## Abstract

Individuals who use opioids have higher rates of hospitalization compared to the general population. Insufficiently treated withdrawal and pain are major factors contributing to high rates of self-initiated hospital discharges (also referred to as leaving against medical advice) in this population. While injectable opioid agonist therapy is limited or unavailable in the majority of Canadian communities, intravenous hydromorphone (IV HM) is widely available in the hospital setting and high-dose IV HM may be a useful treatment adjunct to improve comfort and engagement in inpatient care for some individuals who use opioids. However, major barriers to its use exist including lack of comfort amongst healthcare providers and hospital policies restricting administration. In this commentary, we highlight the potential usefulness of high-dose IV HM as a treatment adjunct for individuals who use opioids in the hospital setting and advocate for expanded hospital policies to facilitate its use.

## Background

The overdose crisis is one of the most significant and devastating public health challenges in recent memory. The 2019 novel coronavirus (COVID-19) pandemic has generated an additional public health crisis, with growing evidence that COVID-19 has exacerbated the overdose crisis and contributed to increased mortality among people who use drugs [1]. Even during non-pandemic circumstances, people who use drugs  have higher rates of inpatient hospitalization and experience increased rates of self-initiated hospital discharges (also referred to as leaving against medical advice) that can result in suboptimal medical treatment [2]. Despite the provision of opioid agonist therapy (OAT) and oral opioids to manage cravings, pain, and withdrawal in hospital, some individuals will continue to inject drugs or choose to leave hospital in order to do so [2, 3].

In select places in Canada and many places in Europe, injectable opioid agonist treatment (iOAT) is available as part of the spectrum of treatment for opioid use disorder (OUD) [4]. It is recommended that injectable treatment be continued in hospital should an individual on iOAT require hospitalization in order to prevent loss of tolerance [4]. In response to the escalating overdose crisis and increasingly toxic drug supply, “safer supply” prescribing, whereby tablet hydromorphone is prescribed as a pharmaceutical alternative to the illicit drug supply for use by the individual as they desire, which may include injection, has been implemented by some physicians and nurse practitioners in certain Canadian cities and provinces [5, 6]. For those individuals using hydromorphone tablets by injection, offering injectable treatment in hospital is arguably also critical to avoid loss of tolerance and provide comfort. Further, there are some people who use opioids who will reject oral OAT and may benefit from the provision of injectable opioids in hospital. Despite being widely available on hospital formularies, the use of IV HM is largely restricted in many Canadian hospitals, with limits on maximum dosing and administration frequency. Similar restrictions likely exist in many countries. Accordingly, we advocate that the use of high-dose intravenous hydromorphone (IV HM) in hospital represents an under-utilized solution to promote engagement in care and enhance satisfaction for certain individuals who use opioids. This requires the adaptation of hospital-level policies and enhanced education to facilitate the use of IV HM in the inpatient setting.

## Community-based injectable hydromorphone

Injectable opioid agonist treatment (iOAT) with pharmaceutical-grade heroin (diacetylmorphine) or high-dose IV HM has emerged as an evidence-based treatment for select individuals with OUD, shown to reduce non-prescribed opioid use and associated crime, overdose risk, and medical consequences of unsafe injection practices [4]. While iOAT may serve as a means to engage individuals in substance use treatment who may otherwise not have been able or willing to do so, community-based iOAT is only available in a few jurisdictions in Canada. Limiting factors relate to the high cost of service delivery as well as regulatory and governmental restrictions, which have remained relatively unchanged despite growing calls for iOAT expansion. Given the pressing need for expansion of safer alternatives to the illicit drug supply given mounting overdose deaths, support for safer supply prescribing as a harm reduction measure has grown; the province of British Columbia recently released guidelines for such prescribing and several safer supply programs have been federally funded in Canada [6, 7]. It is therefore likely that growing numbers of individuals who use prescribed opioids by injection may be seen in the hospital setting.

## The role for high-dose IV HM in hospital

Inadequate pain and withdrawal management, in addition to perceived stigma from healthcare providers, are significant factors cited by people who use drugs  who choose to self-initiate discharge [2, 3]. Pain control and withdrawal management is often achieved with the provision of first-line OAT such as buprenorphine/naloxone or methadone, as well as with short acting opioids and non-opioid adjuncts for additional comfort. While this is adequate for many patients who use opioids, a subset of individuals will choose to continue using non-prescribed IV opioids in hospital, which poses an increased risk of overdose, or to self-initiate discharge, which is associated with increased mortality [8]. Importantly, COVID-19 brings additional concerns of community spread for those who are under investigation for the virus or confirmed positive. Hospitals are uniquely positioned to offer IV HM given that many of the essential elements of such a program already exist including the ability for post-dose monitoring, availability of high concentration HM, and facilitation of IV access.

For individuals in community iOAT programs, ongoing prescribing of high-dose IV HM would be facilitated in hospital, once stable, to prevent loss of tolerance as detoxification carries significant risk of mortality [4]. However, there are several additional circumstances whereby high-dose IV HM may enhance inpatient care for some individuals who use opioids. First, similar to those on iOAT, individuals receiving safer supply hydromorphone may also be using prescribed opioids intravenously, although, since dosing is not observed, caution is needed to ensure adequate tolerance before administration. While transition to oral treatment would be offered, ongoing use of IV HM may be the patient’s preference and, particularly if they are not interested to switch to oral OAT, would help prevent loss of tolerance and facilitate transition back to the community upon discharge. Second, high-dose IV HM can be used as an adjunct during OAT titration—particularly in the case of methadone—as titration to a therapeutic dose can take several weeks. While supplementation with oral opioids is typically adequate during OAT titration, some individuals may prefer, or feel that they require, IV treatment for symptom management. As mentioned, there are also individuals who are not interested to engage in OAT while in hospital and choose either to continue non-prescribed opioid use in hospital or self-discharge if their symptoms are not adequately managed. In such cases, high-dose IV HM may help to foster engagement and allow them to complete their medical treatment, thereby reducing re-admission and associated morbidity [8, 9]. Lastly, in the context of COVID-19, high-dose IV HM may have additional benefits of preventing community or hospital spread by allowing an individual to remain in their room under isolation. A recent guidance document released by the Canadian Research Initiative in Substance Misuse (CRISM) endorses the use of subcutaneous or intravenous opioids for patients in the hospital for whom oral options are not sufficient [10]. Despite this recommendation, significant changes have not occurred in many hospitals that would allow for this to be readily facilitated.

## Considerations and barriers to high-dose IV HM in hospital

The use of high-dose IV HM in hospital does warrant careful consideration and appreciation of potential associated risks. While there are published iOAT guidelines for the outpatient setting, there are no guidelines to support the management of inpatient IV HM, which makes implementation more challenging for providers unfamiliar with this treatment [4]. There is the potential for iatrogenic overdose, although this could be mitigated by post-dose monitoring and cautious titration within the constraints of possible nursing assessment. There may be concerns that high-dose IV HM may reduce engagement in evidence-based OAT treatment and increase overdose risk as it cannot be continued on discharge in the majority of cases. However, some PWUD will choose not to use OAT regardless or wish to remain in a safer supply program. Offering such a strategy in this circumstance alongside the full spectrum of addiction care and OAT respects individual autonomy and the rights of PWUD from a harm-reduction perspective.

Despite national recommendations to consider escalation to IV opioids in the inpatient setting for select individuals who use opioids, some hospitals’ currently established policies may present a major barrier to the practical implementation of high-dose IV HM. These include restrictive limitations on the maximum dose of IV HM that can be administered on medical wards, where the majority of people who use opioids in hospital would be located. While additional training of pharmacy and nursing staff may be needed to facilitate safe implementation, protocolization of high-dose IV HM such as through the use of standardized order sets can provide safeguards for prescribing and administration [9]. As inpatient addiction medicine consult teams continue to expand in many countries including Canada, expertise to support this type of prescribing in select circumstances will likely continue to grow.

## Conclusions

While suboptimal symptom management and stigma from healthcare providers have negatively impacted the inpatient care of people who use drugs  for many years, the current landscape of the overdose crisis, ubiquity of fentanyl within the illicit drug supply, and the occurrence of COVID-19 are all factors generating a pressing need to expand inpatient opioid management strategies. For certain individuals who use opioids, the use of IV HM may help to enhance patient engagement and comfort while in hospital and reduce self-initiated discharge. In consultation with prescribers with expertise in this area, hospitals must revise their policies in order to facilitate administration of high-dose IV HM if felt to be indicated. Widespread expansion of iOAT as a community treatment modality and other alternatives to the toxic drug supply are also urgently needed.

## Data Availability

Not applicable.
